# Cancers primitifs de la verge: à propos de 11 cas et revue de la littérature

**DOI:** 10.11604/pamj.2018.31.14.13077

**Published:** 2018-09-04

**Authors:** Houyem Mansouri, Ines Ben Safta, Med Ali Ayadi, Selma Gadria, Tarek Ben Dhiab, Khaled Rahal

**Affiliations:** 1 Service de Chirurgie Carcinologique, Institut Salah Azaiz de Tunis, Tunisie

**Keywords:** Cancer de la verge, carcinome épidermoide, chirurgie, circoncision, Penile cancer, squamous cell carcinoma, surgery, circumcision

## Abstract

Le cancer de la verge est une pathologie rare en Tunisie ou la circoncision est de pratique courante. Il s'agit dans 95% des cas d'un carcinome épidermoïde. Le traitement repose essentiellement sur la chirurgie. Nous rapportons rétrospectivement les caractères épidémiologique, clinique, thérapeutique et évolutif de 11 cas de cancers primitifs de la verge.

## Abstract (en)

Penile cancer is a rare pathology in Tunisia where circumcision is a common practice. Squamous cell carcinoma accounts for 95% of cases. Treatment is essentially based on surgery. We conducted a retrospective study in order to highlight the epidemiological, clinical, therapeutic features as well as the evolutionary characteristics of 11 cases with primary penile cancer.

## Introduction

Le cancer de la verge est une pathologie rare en Tunisie ou la circoncision est de pratique courante. Il s'agit dans 95% des cas d'un carcinome épidermoïde [1]. La tumeur siège au niveau du gland et le prépuce dans 90% des cas et au niveau du corps pénien dans moins de 2% des cas. Le traitement repose essentiellement sur la chirurgie.

## Méthodes

Il s'agit d'une étude rétrospective de 11 cas de cancers primitifs de la verge colligés à l'institut Salah Azaiz sur une période de 18 ans (Janvier 1993-Décembre 2011). Une étude des caractères épidémiologiques, cliniques, thérapeutiques et évolutifs a été faite.

## Résultats

L'âge moyen de nos patients est de 64 ans (40-84 ans). Le délai moyen de consultation était de sept mois. Un seul patient présentait des lésions précancéreuses; il s'agissait de lésions condylomateuses. La tumeur siégeait dans la racine de la verge chez trois patients, le corps de la verge chez deux patients, le gland chez cinq patients et à cheval sur le gland et la verge chez un autre. La taille tumorale moyenne était de 33.5 mm (5 mm à 100mm). On a classé les tumeurs selon le système TNM 2009: un patient au stade T4N2M0, deux au stade T3N0M0, un autre au stade T2N2M0, quatre au stade T1N0M0, et trois au stade T1N2M0. La biopsie pratiquée chez tous nos patients a conclu à un carcinome épidermoïde dans neuf cas et à un sarcome de Kaposi dans deux cas. Sept de nos patients étaient opérés: trois patients ont eu une amputation partielle (AP); un patient a eu une amputation totale (AT) avec des marges saines. Un curage ganglionnaire inguinal bilatéral était pratiqué chez les quatre patients. Deux patients ont eu une exérèse large carcinologique qui était associée à un prélèvement ganglionnaire dans un cas. Un patient a eu un curage ganglionnaire inguinal bilatéral précédé d'une curiethérapie et une radiothérapie externe, un autre patient était traité par contact-thérapie seule. La radiothérapie adjuvante était indiquée chez quatre patients ayant une atteinte ganglionnaire histologique. Deux patients ont eu une chimiothérapie seule. Après un suivie de 60 mois, trois patients ont récidivé avec un délai moyen de récidive de 5.66 mois (extrêmes: 2-10 mois). La récidive siégeait au moignon d'amputation partielle chez un patient et traitée par émasculation et radiothérapie externe, les deux autres patients ont présenté une récidive ganglionnaire inguinale et ilio-obturatrice traités respectivement par radiothérapie externe et chimiothérapie associant la Bléomycine, la cisplatine et la Méthotrexate (Tableau 1).

**Tableau 1:** Principales caractéristiques des patients

p	TNM	Taille Mm	Type TTT	Type histo	Type Chir gg	Marge	N+/N	Récidive siège	Récidive Délais	Récidive TTT
1	T4N2M0	65	AT	C. epider	C IngB	saine	12N+/20N	-	-	-
2	T1N2M0	61	AP	C. epider	C IngB	saine	4N+/16N	Moignon Verge	2 mois	AT+RTE
3	T2N2M0	40	AP	C. epider	C IngB	saine	2N+/13N	Inguinale	5 mois	RTE
4	T3N0M0	78	AP	C. epider	P IngB	saine	10N-	-	-	-
5	T1N2M0	45	CT	C. epider	-	-	-	-	-	-
6	T1N0M0	72	EX	Sarcome	-	saine	-	-	-	-
7	T1N0M0	84	Curi	C. epider	-	-	-	-	-	-
8	T1N0M0	75	EX	C. epider	P Ing B	saine	2N-	-	-	-
9	T1N2M0	50	RTE+ curi	C. epider	C IngB	-	2N+/8N	Ilio-obturatrice	10 mois	CT
10	T3N0M0	72	-	C. epider	-	-	-	-	-	-
11	T1N0M0	65	CT	Sarcome	-	-	-	-	-	-

CT: chimiothérapie, RTE: radiothérapie externe; AT: amputation totale, AP: amputation partielle, EX: exérèse, CIngB: curage inguinal bilatéral, P IngB: prélèvement inguinal bilatéral, curi: curiethérapie, C. epider: carcinome épidermoïde, chir gg: chirurgie ganglionnaire, TTT: traitement

## Discussion

Le cancer de la verge représente 0.5% des cancers chez l'homme. L'âge moyen de survenu est de 50 ans [1], l'âge moyen de nos patients est de 64.27 ans. Il s'agit le pus souvent de tumeurs distales. Le carcinome épidermoïde représente le type histologique le plus fréquent dans 95% des cas, les mélanomes et les sarcomes sont plus rares [1]; deux de nos patients avaient un sarcome de Kaposi. Plusieurs facteurs de risque ont été rapportés dans la littérature dont l'hygiène défectueuse, le phimosis d'où le rôle protecteur de la circoncision, certaines infections virales telles que l'HPV ainsi que les lésions précancéreuses telles que le lichen scléreux, le condylome et la maladie de Bowen [2]. Dans notre série, un seul patient était suivi pendant deux ans pour des lésions condylomateuses de la verge et deux patients étrangers n'étaient pas circoncis. Tous nos patients ont eu une biopsie profonde de la lésion. Cette biopsie n'est pas nécessaire s'il n'existe pas de doute diagnostique. En cas de réalisation de ce geste, une biopsie-excision avec des marges saines est préférable à une simple biopsie [3]. Le bilan d'extension locorégionale dans notre série repose sur l'examen clinique et l'échographie. Le traitement conventionnel du cancer de la verge reposait sur l'amputation partielle ou totale de la verge associée ou non à un curage ganglionnaire ilio-inguinal bilatéral permettant d'assurer un contrôle local dans 90% des cas [4]. Cette chirurgie mutilante est souvent refusée par nos malades ce qui a motivé les procédures de conservation d'organe incluant les exérèses larges, la technique de Mohs, la radiothérapie externe, la curiethérapie, le laser et la chimiothérapie. Bien que ces moyens ne semblent pas assurer le même contrôle local comparé à la chirurgie radicale, ils représentent des options thérapeutiques recommandées pour les stades précoces (Tis, Ta, T1) de bas grade. Pour les tumeurs T1 < 2cm de grade 1 à 2, une exérèse assurant une marge de sécurité de 0.5 à 1cm est recommandée [5]. Les tumeurs T1 de grade 3-4 ou T >= 2 exigent une chirurgie plus extensive reposant sur une amputation totale ou partielle de la verge selon la taille tumorale et la profondeur de l'invasion [6]. Toutefois une chirurgie conservatrice peut être proposée si l'envahissement tumoral ne dépasse pas la moitié du gland chez des patients pouvant coopérer avec une surveillance stricte vue le risque élevé de récidive en l'absence de chirurgie radicale. Ce principe semblerait contradictoire avec les résultats de la série de 127 cas du Memorial Sloan-Kettering Cancer Center (MSKCC) publiés par Moses *et al* en 2014 et qui rapportent un une survie spécifique à 3 ans de 100% en cas biopsie exérèse, de 71% en cas d'AP et de 57% en cas d'AT [7]. Ces résultats ne seraient pas liés à l'étendu de la chirurgie mais plutôt au fait que les patients ayant eu une chirurgie conservatrice étaient diagnostiqués à des stades TNM précoces.

Dans une étude de 700 cas de (CEP) menée par Leijte JA *et al*, 205 récidives étaient rapportées après une rémission de 5 ans dans 92.2% des cas. Le taux de récurrence était de 27.7% en cas de traitement conservateur et de 5.3% en cas d'amputation [8]. Bien que les récidives locales ne semblent pas augmenter le risque de métastases ganglionnaires, leurs survenue après un traitement conservateur diminue la survie spécifique ce qui suggère que les récidives suite à une chirurgie conservatrice reflètent plutôt l'agressivité des tumeurs [9]. Dans notre étude le taux de récurrence était de 27.27% (trois patients) dans un délai moyen de 5.66 mois: deux patients avaient récidivé après une amputation partielle et un patient après un traitement conservateur associant une curiethérapie, une radiothérapie externe et une chirurgie ganglionnaire. Devant le refus habituel de l'amputation, la curiethérapie interstitielle après circoncision constitue une alternative intéressante. Le contrôle local par curiethérapie est de 70% à 90% pour les tumeurs de moins de 4 cm (T1-2) avec plus de 90% de conservation d'un pénis fonctionnel [10]. Dans notre série, un patient était traité par curiethérapie après circoncision pour une lésion du sillon balano-prépucial de 25mm et n'a pas récidivé, un deuxième patient était traité par radiothérapie externe et curiethérapie associées à un curage ganglionnaire bilatéral mais a présenté une rechute ganglionnaire ilio-obturatrice après 10 mois. En ce qui concerne la prise en charge ganglionnaire, on a réalisé un curage inguinal bilatéral chez quatre patients classés cN2 ,ce qui est recommandé par certaines équipes et qui peut être curateur [11]. Les ganglions étaient envahis dans tout les cas .Deux patients classés cN0 avaient eu un prélèvement ganglionnaire bilatéral avec absence d'envahissement tumoral (Tableau 2). En l'absence adénopathies palpables, la prise en charge dépend des facteurs de risque d'extension ganglionnaire représentés essentiellement par le stade, le grade et l'invasion lympho-vasculaire [12]. De ce fait, l'association européenne d'urologie a défini trois groupes de risque: bas risque (Tis, Ta, T1a ), risque intermédiaire (T1b) et haut risque (T2 ou G3 ou G4) [13]. La simple surveillance est recommandée chez les patients de bas risque vue que le risque de micro-métastases occultes n'est que de 17% [14]. Pour le groupe de haut risque et de risque intermédiaire, une lymphadénectomie inguinale radicale ou modifiée est recommandée et doit être bilatérale vue l'impossibilité de prédire le risque d'atteinte inguinale controlatérale en se basant sur le siège tumoral [13]. Dans la série publiée par Niels *et al* [12], 77% des curages ganglionnaires pratiqués chez les patients de haut risque étaient négatifs d'où la place du ganglion sentinelle permettant de diminuer la morbidité élevée de ce geste qui est de l'ordre de 30 à 70% en cas de curage radical et de 3.4 à 6.8% en cas de lymphadénectomie modifiée [15]. Le nombre réduit de cas dans les séries et l'absence de randomisation ne permettent pas de déterminer l'impact de la chimiothérapie néo-adjuvante ou adjuvante sur la survie et le contrôle local. Toutefois, elle est recommandée en cas de tumeur pN2-3 ou de masse inextirpable ou de récidive ganglionnaire [16].

**Tableau 2:** Résumé de l’atteinte ganglionnaire clinique et histologique

	Examen des AG	Type chirurgie gg	Atteinte gauche N+/R+/N	Atteinte droite N+/R+/N
P1	ADP suspectes bilatérales	Curage bilatéral	1N+/R-/3N	1N+/R-/5N
P2	Masse inguinale droite de 3cm ADP gauches suspectes gauches	Curage bilatéral	12N+/R-/20N	Magma de gg N+
P3	ADP suspectes bilatérales	Curage bilatéral	2N+/2R+/11N	2N+/2R+/5N
P4	ADP inguinales unilatérales gauches	Curage bilatéral	2N+/R-/8N	5N-/5N
P5	ADP inguinale unilatérale gauche	Prélèvement bilatéral	5N-/5N	5N-/5N
P6	Aires ganglionnaires libres	Prélèvement bilatéral	1N-/N	1N-/1N

AG : aires ganglionnaires, ADP : adénopathie, R+ : rupture capsulaire, R- : absence de rupture capsulaire, N+ : ganglion métastatique, N- : ganglion indemne

## Conclusion

La faible incidence des cancers de la verge représente un obstacle à la publication de séries conséquentes de patients, permettant de codifier la prise en charge thérapeutique. La circoncision semble avoir un rôle protecteur. Le pronostic dépends de la profondeur d'infiltration, du grade histo-pronostique de l'atteinte ganglionnaire et de la précocité de la prise en charge. La chirurgie d'exérèse éventuellement associée à un curage ganglionnaire en fonction du stade reste le traitement de référence.

### Etat des connaissances actuelle sur le sujet

- Pathologie rare;
- Le carcinome épidermoide est la forme la plus fréquente;
- La chirurgie est le traitement de référence.

### Contribution de notre étude à la connaissance

- La circoncision pourrait avoir un role protecteur;
- La chirurgie conservatrice est possible;
- Les indications du curage ganglionnaire.

## Conflits d’intérêts

Les auteurs ne déclarent aucun conflit d'intérêts.
